# Liposomal Vincristine as a Bridge Therapy Prior to CAR-T Therapy in Relapsed and Refractory Diffuse Large B-Cell Lymphoma?

**Published:** 2019-04-01

**Authors:** Juskaran Chadha, Shafinaz Hussein, Yougen Zhan, Jonah Shulman, Joshua Brody, Lynn Ratner, Amir Steinberg

**Affiliations:** 1 Department of Hematology & Medical Oncology, Lenox Hill Hospital Northwell Health, New York, NY, USA; 2 Tisch Cancer Institute, Mount Sinai Hospital, New York, NY, USA

**Keywords:** CAR-T, Liposomal vincristine, DLBCL, Heavily pretreated, Autologous SCT

## Abstract

We report a case of a 76-year-old male with a history of relapsed and refractory diffuse large B-cell lymphoma (DLBCL).Our patient was initially treated with front line chemotherapy along with central nervous system (CNS) prophylaxis with complete response. He subsequently relapsed, was sensitive to second-line chemotherapy, and underwent autologous stem cell transplantation achieving a complete remission. Only a few months after transplant, the patient suffered his second relapse and was deemed a candidate for Chimeric Antigen Receptor T-Cell Therapy (CAR-T). Given his aggressive disease, combined with the time needed to generate CAR-T cells, a multidisciplinary team recommended to treat our patient with liposomal vincristine in combination with rituximab as a bridge therapy. Durable responses have been seen using liposomal vincristine based on results from a recent phase II trial in heavily pretreated patients with DLBCL^1^. This therapy was effective in stabilizing and reducing active disease in our patient. This case looks to illustrate the use of liposomal vincristine in combination with immunotherapy in a novel setting bridging highly selected patients with active and refractory lymphoma prior to CAR-T. Moreover, we expanded an additional therapeutic point, highlighting the importance of optimal disease control prior to CAR-T cell harvesting, as recent literature has shown that residual malignant cells in the pheresis product may be inadvertently be transfected with the CAR gene, resulting in resistance and further relapse^2^.

## Introduction

 In the heavily pretreated population of DLBCL, CAR-T is an additional therapeutic option in our armamentarium. Briefly, we describe a case of an aggressive DLBCL and the use of liposomal vincristine for controlling the primary disease and bridging the patient to CAR-T. 

## Case presentation

This case report is a summary of the clinical course of a 75-year-old male who was initially diagnosed to have DLBCL with concomitant Hemophagocytic Lymphohistiocytosis (HLH). PET/CT was abnormal with increased uptake in bulky nodes in the celiac, portal, and aortocaval nodes. Celiac lymph node biopsy was interpreted as a B-cell lymphoma with Ki-67 of 52-60%. The patient also had a bone marrow biopsy performed, showing B-cell lymphoma of intermediate to large sized B cells, FISH was negative for MYC/BCL2/BCL6 rearrangements. For the diagnosis of HLH, our patient received etoposide and dexamethasone per HLH-94 protocol with good response. The patient completed 6 cycles of R-CHOP-based chemotherapy for DLBCL. Unfortunately, 9 months after completing treatment, our patient had a clinical relapse, including rising WBC and new and worsening generalized adenopathy. He was subsequently prescribed salvage cytotoxic chemotherapy with sensitive response and was planned for autologous stem cell transplant with inpatient high-dose chemotherapy with stem cell rescue therapy for primary relapsed disease. The Patient engrafted by day +9 and had no residual disease on repeat imaging and stable blood work. However, 2 months after engraftment, patient was found to have both laboratory and clinical relapse, including a white blood cell (WBC) greater than 30K with 73% blasts in the peripheral blood, a platelet count of 42, and a positive bone marrow biopsy with aberrant B cell population of 54-58% and Ki-67 of 70-80%. Given our patient’s refractory and relapsed disease after autologous stem cell transplant, he was considered a candidate for CAR-T therapy. 

**Figure1:**
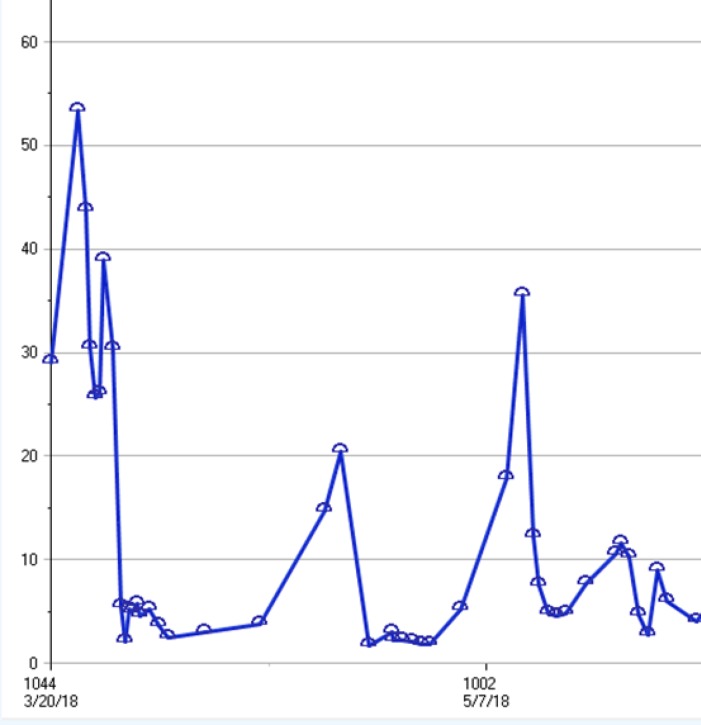
Blood work following each infusion, suggesting reduction in overall WBC

In order to control his disease prior to harvesting cells for CAR-T therapy, the patient was recommended to start systemic therapy with liposomal vincristine and rituximab. Patient was able to tolerate 4 cycles of treatment administered intravenously every 2-3 weeks without significant adverse effects. Follow-up blood work and flow cytometry after each infusion suggested chemo sensitivity with a reduction in overall WBC and blast count, without significant myelosuppression (Figure 1).

**Figure:**
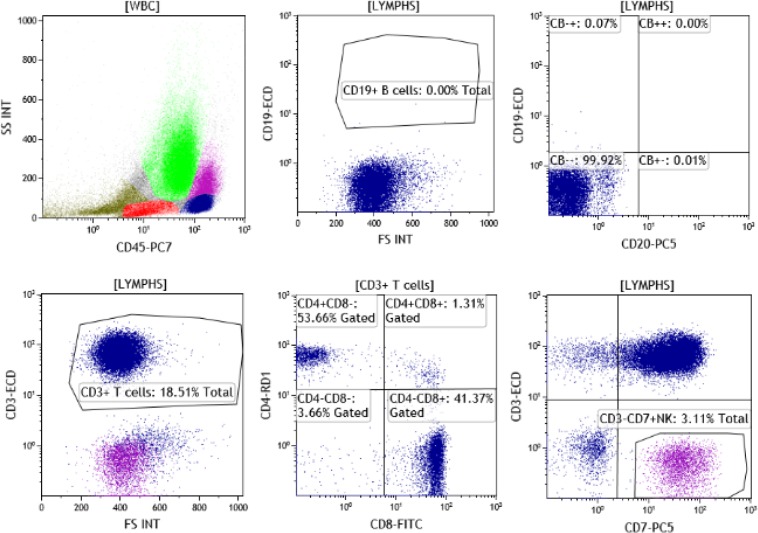


Figure 2 illustrates the flow cytometric analysis of the peripheral blood after rituximab and liposomal vincristine therapy. The analysis shows a vast majority of T-cells with CD4:CD8 ratio of 1.3. There are no B cells. 

**Figure:**
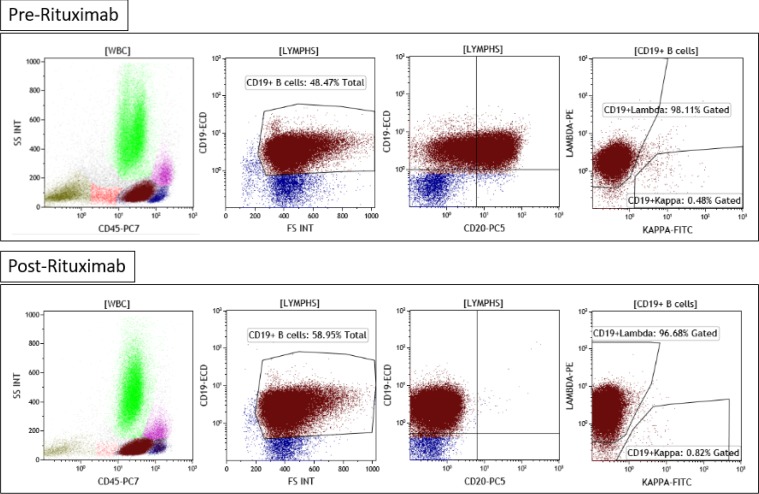


In Figure 3, the bone marrow flow cytometric analysis is shown. Because our patient had received multiple prior lines of therapy with rituximab and then demonstrated a CD20(-) phenotype, we expected that the majority of the anti-lymphoma effect observed was attributed to the VSLI therapy. In subsequent follow-up, our patient presented with a left facial droop and evaluation for CNS disease was positive. He received treatment for CNS involvement with two doses of intrathecal methotrexate and proceeded to enrollment in CAR-T trial. Unfortunately, he was refractory to CAR-T therapy despite multiple attempts. As further salvage, he received additional doses of liposomal vincristine with rituximab; however, with short duration of response was evaluated for hospice and ultimately succumbed to his aggressive disease. 

## Discussion

 Vincristine Sulfate Liposome Injection (VSLI) was originally FDA approved in 2012 for the treatment of Adult Acute Lymphocytic Leukemia (ALL) in patients with second or greater relapse and clinically advanced Philadelphia chromosome negative ALL^3^**.** Additional data in the literature has been published in 2014, showing utility of VSLI and Rituximab in patients with relapsed and refractory diffuse large B-cell lymphoma (DLBCL) or Mantle Cell Lymphoma (MCL) in need of palliative therapy^1^. 

In adult patients with ALL, despite high complete remission rates, relapses are unfortunately common^4^. At present, there is no standard in regards to salvage therapy and the only potentially curative option would be allogeneic HSCT^5^. Ultimately, these patients’ outcomes are worse with a CR rate of 18% seen in retrospective analysis^6^. Given the futility of treatment with no standard of care in the relapsed setting, VSLI as a single agent was given FDA accelerated approval based on a relatively small phase II clinical trial^7^. The concept behind the treatment is to allow for safe delivery of increased dose of vincristine using a liposomal package, as opposed to traditional Vincristine which would result in significant neurotoxicity at higher doses and myelosuppression^3^. The similarity of liposomes to cell membranes allows for the drug to be effectively delivered to target regions with improved bioavailability^8^^,^^9^^,^^10^^,^^11^. The liposomes are composed of phospholipid sphingomyelin and cholesterol^13^^,^^14^. Moreover, the injection exhibits slower systemic release and better penetration to organs and bone marrow compared with standard vincristine, without suppressing the bone marrow^10^^,^^13^^,^^14^^,^^15^^,^^16^^,^^17^. 

In nonclinical tests, VSLI had a greater maximum tolerated dose than standard vincristine and demonstrated superior anti-lymphoid cancer activity without additional toxicity ^13^^, ^^18^^, ^^19^^, ^^20^^, ^^21^. The basis for accelerated approval for VSLI in 2012 came from a multinational, phase II, single-arm trial of 65 patients. Heavily pretreated patients with advanced B or T-cell lineage Ph negative ALL received weekly monotherapy dosing. The OR rate among the 65 patients treated with VSLI was 35% with 20% CR and median OS of 4.6 months^22^**. **Importantly, toxicity was predictable and manageable despite the delivery of large normally unachievable doses of drug^3^**. **

VSLI’s enhanced penetration and concentration in tissues including non-Hodgkin lymphoma (NHL) target tissues prompted further investigation in NHL patients^1^ In a phase II study of advanced pretreated NHL, single agent VSLI 2.0 mg/m2 every 2 weeks resulted in an OR of 25% with aggressive histology^23^. Given these results, an additional phase II study was published in 2013 which reviewed 22 patients with heavily pretreated NHL including CD20+ DLBCL or mantle cell lymphoma in need of palliative therapy, single arm, and open-label study of VSLI 2.0 mg/m2 without dose cap with rituximab. Doses of VSLI were administered as 1-hour infusions, every 2 weeks up to 12 doses plus 4 weekly doses of intravenous rituximab 375 mg/m2. The median number of cycles was 5 (range, 2-12). Objective response rates were seen in 59% of patients, including complete response in 27%. The median response duration was 147 days. Regarding safety, there were no treatment-associated mortalities or unexpected toxicities. There were only four patients who experienced a grade 4 adverse event, neutropenia or leukopenia. There was no grade 4 neuropathy. Overall, therapy was generally well tolerated with a more favorable overall response rate compared to VSLI monotherapy and further dose intensification compared to traditional vincristine^1^**. **

Chimeric Antigen Receptor (CAR) T-Cell Therapy was FDA approved in 2017 for patients with refractory DLBCL, Axicabtagene Ciloleucel (Yescarta) based on ZUMA-1 phase 1-2 multi-center trial. In 2018 a second CAR-T therapy was approved for lymphoma, Tisagenlecleucel (Kymriah) based on JULIET phase 2 clinical trial. CAR-T immunotherapy involves engineering patients’ own white blood cells. These T-cells are genetically modified to recognize cancer cells, particularly cells expressing CD19 on their surfaces. Moreover, as a backbone of therapy, the T cells play a major role in mobilizing the immune system and killing pathogens^24^**. **In a recently published CAR-T trial in patients with CD19+ DLBCL, eligibility criteria included patients with measurable disease after primary and salvage therapies. Additional criteria included no curative treatment options and limited overall prognosis. It is important to note that there was no standardized bridging therapy prior to initial leukapheresis. In the trial, they addressed that after initial leukapheresis, patients could receive bridging therapy at the discretion of the primary physician, of which 10 of 28 patients received prior to lymphodepleting chemotherapy^25^. However other CAR-T trials, including ZUMA-1, have not allowed systemic bridging chemotherapy after leukapheresis and prior to lymphodepleting chemotherapy or before infusion of cells^26^**. **It should be noted that in the latter study, there was a short turnaround time between leukapheresis and infusion of cells of 17 days. 

This clinical case report supports an innovative role for therapies such as VSLI in combination with rituximab prior to T cell harvesting and/or enrollment in a CAR-T trial. Clinically, it is a delicate balance to avoid further morbidity and mortality while treating patients who are being evaluated for CAR-T therapy prior to leukapheresis so that patients maintain an optimal performance status. In the case of our patient, his leukemic variant of relapsed and refractory DLBCL was extremely advanced including increased peripheral blasts, systemic bulky disease, and CNS involvement. This prompted the need to treat the patient while maintaining quality of life, performance status, and eligibility for CAR-T trial therapy. In more frail patients, or ones with more manageable disease, we could consider additional debulking agents including bendamustine, as well as other milder therapies. 

Based on recently published data, the need for optimal disease control prior to CAR-T pheresis may play a significant role in the efficacy of its therapy. A recent report showed that residual malignant cells in the CAR-T pheresis product can inadvertently be transfected with the CAR gene making them resistant to CAR-T killing and promote relapse. The case in this recent publication described a young male with B-ALL and refractory disease enrolled in a phase 1 trial using anti-CD19 chimeric antigen receptor T cell product. He underwent lymphodepletion followed by infusion of the CAR-T cells. At nine months the patient was found to have a frank relapse. It was elucidated that the transduction of one leukemic cell containing anti-CD19 CAR during manufacturing was able to breed resistance to CAR-T therapy via binding to the CD19 leukemic cells in cis formation, concealing the CD19 epitope, and revealing a CD19 negative leukemic population via immunophenotyping^2^. Based on this rare phenomenon, it is paramount to improve in vivo purging of malignant cells. In that regard, we were impressed for our patient by the drastic and rapid elimination of greater than 99.99% of malignant B cells within days of therapy with intact CD4 and CD8 T cell counts amenable to pheresis for CAR-T production (Figure 2). This paper highlights the unique role for VSLI therapy in an advanced patient population. With the ultimate goal of being eligible for CAR-T therapy, and achieving a durable complete response, VSLI along with immunotherapy may safely get patients into initial CAR-T evaluation. Based on recent literature, additional therapeutic benefit may also be derived with bridging therapy to fully eliminate residual tumor cells in the periphery, thus minimizing contamination, optimizing manufacturing practices, and reducing the risk of resistance in CAR-T therapy. Lastly, perhaps the treatable patient population could be expanded to include select cases with refractory ALL, since ALL is what VSLI was initially FDA approved for and there is an FDA approved CAR-T therapy for ALL, Tisagenlecleucel (Kymriah). 
